# Spatially Resolved Analysis of Urban Thermal Environments Based on a Three-Dimensional Sampling Algorithm and UAV-Based Radiometric Measurements †

**DOI:** 10.3390/s21144847

**Published:** 2021-07-16

**Authors:** Daniel Rüdisser, Tobias Weiss, Lukas Unger

**Affiliations:** 1 Buildings Department, AEE Institute for Sustainable Technologies, 8200 Gleisdorf, Austria; 2 Skyability GmbH, 7011 Gemeinde Siegendorf, Austria; Lukas.unger@Skyability.com

**Keywords:** thermal comfort, urban heat islands, mean radiant temperature, drone imaging, thermal radiation, global radiation, radiometry, perceived temperature, 3D model processing

## Abstract

A new method and workflow to assess outdoor thermal comfort and thermal stress in urban areas is developed. The new methodology is applied to a case of an urban quarter in the city of Graz. The method recognises the significance of detailed and accurate spatially resolved determination of mean radiant temperatures taking into account all relevant radiative components, comprising thermal radiation, as well as global radiation. The method relies on radiometric imaging data that are mapped onto a three-dimensional model. The image data are acquired by means of drones (UAVs) equipped with multispectral and thermographic cameras to capture short- and long-wave radiation. Pre-existing city models and a Monte Carlo raytracing algorithm to perform anisotropic sampling based on a 3D model with human topology are used to determine local radiation temperatures with high spatial resolution. Along with spot measurements carried out on the ground simultaneously, the spatially resolved and three-dimensionally determined mean radiation temperatures are used to calculate thermal comfort indicator maps using UTCI and PMV calculation. Additional ground measurements are further used to validate the detection, as well as the entire evaluation process.

## 1. Introduction

Amid climate change and increasing comfort needs, the topic of indoor and outdoor thermal comfort is rapidly gaining in importance. However, the term “comfort” is somewhat misleading or inadequate as situations linked to very high or very low thermal comfort index values are objectively associated with physiological stress. Such environments are not a matter of subjectively perceived discomfort, but can cause serious health problems and increase mortality risks.

A metastudy conducted by the WHO Europe [1] analysed the impacts and influencing factors of heatwaves and recommended climate-relevant planning and building processes to reduce urban heat island effects. For efficient and targeted optimisation of hotspot areas, i.e., intra-urban-heat islands (IHUIs), a comprehensive understanding of the complex interactions and identification of the main influencing factors are necessary. Various simulations tools are increasingly used for this task.

Often based on the pioneering approach formulated in the 1970s by Fanger [2], semi-empirical models are used to calculate thermal comfort indices. A key input parameter for the models is the mean radiant temperature MRT. The level of detail regarding the determination of this parameter varies significantly based on the models used. Commonly known approaches or tools are currently CityComfort+ [3], EnviMet [4], RayMan [5], SOLWEIG [6], Rakha [7] and TownScope [8].

In order to achieve a comprehensive, precise simulation of all urban surface temperatures, it would be necessary to solve an extensive system of differential equations. Additionally, a large number of boundary conditions and material parameters defining the thermal properties of all objects involved (buildings, soils, air body, etc.) has to be defined as accurately as possible. Based on the boundary conditions of solar radiation, air temperature, humidity, wind speed, wind direction, precipitation, cloudiness and additional heat sources, a very complex system of heat transfer unfolds. An accurate dynamic simulation of urban areas requires modelling of all heat transfer mechanisms involved (radiation, convection, conduction) with a sufficiently high spatial and temporal resolution and over a reasonably large area.

Therefore, due to the complexity of the processes and objects involved, it is challenging to predict absolute values by means of simulation tools. However, these simulation methods are well suited for the analysis of relevant sensitivities and interactions. Various numerical studies, primarily based on EnviMet simulations, have shown that typical urban geometries have a significant impact on the microclimate and thermal comfort within urban environments (see, e.g., Krüger et al. [9] Deng and Wong, 2020 [10], or Ali-Toudert and Mayer, 2006 [11]). Often, metrics such as street aspect ratio and sky view factors are used to analyse and demonstrate the significance of effects related to the three-dimensionality of urban structures in a simplified way. Therefore, it is essential to provide a suitable measurement method that can capture the three-dimensional character of the urban environment to validate simulation results and provide data for enhanced modelling.

In the field of urban outdoor thermal comfort, publications based on simulation methods significantly outnumber empirical studies. Precise in situ measurements of the physical quantities required for thermal comfort evaluation are elaborate and challenging (see, e.g., Thorsson et al., 2007 [12], or Höppe and Mayer 1987 [13]). Hence, it is impossible to apply spot measurement methods to cover an extended urban area with high spatial resolution. We present a method that is able to provide spatially resolved thermal comfort information by performing a multitude of in situ measurements numerically in a digital twin environment. The empirical data necessary to model this environment is captured using cameras on drones and a few ground measurements. 

This publication is an extended version of a conference paper [14,15] presented at the 8th Conference of IBPSA Germany and Austria—BauSIM 2020. Significant improvements of the method, especially the processing of multispectral images, as well as additional validation measurements were carried out in this second application of the method and are covered by the present publication only.

## 2. Method

### 2.1. Method Overview

The method proposed in this publication can, to a certain extent, be viewed as a hybrid method. Fundamentally, it is an empirical method based on measurement data, capturing the thermal environment at a specific time. However, numerical methods, used in the simulation tools mentioned above, are applied and refined in our method to determine local comfort values as precisely as possible.

The development of the method was based on the working hypothesis that mean radiant temperature (MRT) is the key influencing factor regarding thermal comfort in summer urban environments. On the one hand, total radiation represents the dominant component in the physiological heat balance in such an environment. On the other hand, parameters related to radiation also show the highest spatial variations. In order to determine this key parameter MRT with high accuracy, we strived to refine the models as much as possible on both sides: on the “receiver side”, a 3D model with human topology and varying surface properties was used to determine the anisotropic directional sensitivity to the incoming radiation; on the “sender side” (environment), the irradiation field, consisting of the thermal radiation of all surfaces, the atmospheric counter-radiation, as well as direct, diffusely reflected and diffusely scattered solar radiation, was modelled based on measurement data and locally evaluated using numerical methods. In the process, the thermal radiation (also referred to as “longwave” in the following) and the solar radiation (also referred to as “shortwave” or “global radiation” in the following) were processed separately, but in a largely identical manner. The measurement data were captured during drone flights, where a large number of images were recorded by two different cameras. These images containing the long- and short-wave radiation information of all visible surfaces (buildings, vegetation, sealed and unsealed ground areas) represent the key input to the process. Additionally, a three-dimensional city model of the target region is required.

### 2.2. Anisotropic Sensitivity to Radiation

**Figure 1:**
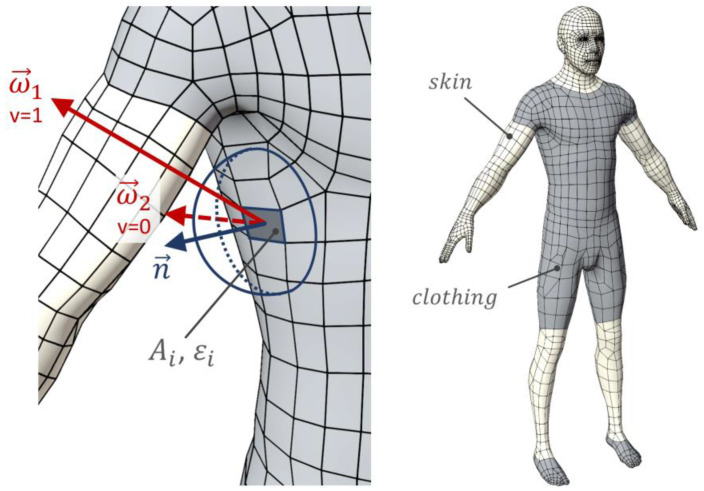
Determination of effective radiation surfaces using a human model.

In order to determine the (anisotropic) angular distribution of the sensitivity to radiation of the human body, a three-dimensional CAD model of a human dummy was used (see Figure 1). The surface of the model was divided into the categories “skin” or “clothing” to assign specific emissivity values *ε* (for longwave radiation), as well as absorption coefficient *α* (for shortwave radiation). For the skin surface, commonly used values were used. For clothed areas, reduced values were used, which correspond better to the average absorption properties of common textiles [16,17]. All values that were used to weight the short- and long-wave absorptions are listed in Table 1.

For the purpose of determining the local specific radiation temperature, considering shortwave and longwave components, it is necessary to evaluate the directional sensitivity distribution, as well as its integral, the effective radiation surfaces *A_eff_*.

To do this, a weighted surface integral over the surface of the human 3D dummy was evaluated. The weighting factors applied represent the “radiative exposure” of the local surface position. They are defined by the product of the absorption coefficients *ε_i_* or *α_i_* with the infinitesimal surface *dA* and a further integral representing the amount of self-shading for each spot, since only directions ω that are not obstructed by the model itself exchange radiation with the environment. In order to determine this value, we define a visibility function v(ω,p) for points p on the surface that has the value 0 for directions obstructed by the model and 1 for non-obstructed directions. Assuming Lambertian surface properties (see Section 2.4), which are a good approximation for the relevant surfaces, a cosine-weighted hemispherical integral must be solved for each point *p* on the subsurface *A_i_*. By the summation over all subsurfaces, we finally yield the total effective radiation surface:

(1) Aeff,lw=∑iεi ∫Ai∫Ωcosθπ·v(ω,p)·dω dA=∫x∫y∫φ=02π∫θ=0π2cosθπ·v(φ,θ,x,y)·sin(θ)dθ dφ dy dx

(2) $$likewise:A_{eff,sw}=\underset{i}{\sum }\alpha_{i}\ldots$$

The difference between the two integrals *A_eff,lw_* and *A_eff,sw_* is determined by the differences regarding the assigned absorption coefficients *ε_i_* and *α_i_*.

The multiple integral defined was solved by means of numerical Monte Carlo integration (sample size: 5E9).

In most common methods [6,18], the anisotropy caused by the human topology is only taken into account for direct solar radiation. However, in our approach, we considered anisotropic sensitivities for all radiative components, including long- and short-wave diffuse radiation. The required angular sensitivity distributions can be determined based on the model just described.

Based on the principle of reversibility, one can imagine the surface of the human dummy as being a diffuse emitter. The radiant exitance of each subsurface would then be defined by the absorption coefficients *ε_i_* (longwave) or *α_i_* (shortwave). The two-dimensional sensitivity function *S*_2*D*_(*φ*,*θ*) corresponds to the normalised angular distribution of the irradiance emitted by the model at infinite distance. If the angular distribution is not normalised, we obtain the distribution *P*_2*D*_(*φ*,*θ*), which corresponds to the projected surface of the dummy for each direction (having the units m^2^).

Since only the direction and not the actual origin point of the individual rays is taken into account, this distribution corresponds to the far-field at an infinite distance. However, since the distribution converges quickly with distance, the function can be used for shorter distances as well.

**Figure 2:**
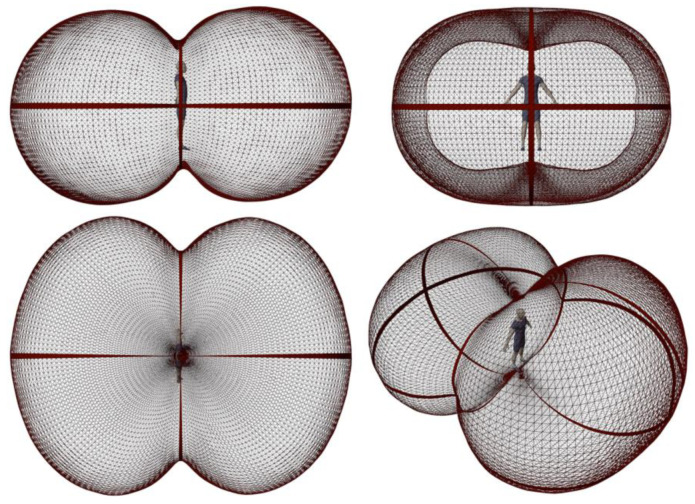
Three-dimensional polar plot of the anisotropic sensitivity/sampling function *S*_2*D*,*sw*_(*φ*,*θ*).

The resulting functions form a peanut-like shape in polar plot representation. The shape is a result of the human topology and the surface weighting factors used (see Table 1). Although the area weighting in the form of the *ε_i_* and *α_i_* coefficients varies, the general shape of the two sensitivity functions (LW/SW) is very similar. Therefore, only the shortwave function is depicted as a polar plot in Figure 2. The shape shows that the sensitivity to thermal radiation is highest towards the front and the backside of the body, whereas the smallest sensitivity can be found towards the top and bottom directions, as the projected surface area is minimal there.

For practical purposes, the use of the derived two-dimensional functions is unsuitable, as the mean radiation sampled with this function would only be valid for a specific azimuthal (compass) orientation of a person. A more practical and general approach is achieved by averaging the distribution with respect to the azimuthal angle *φ*. In this case, it describes the mean value for a large set of randomly oriented persons. Consequently, only these generalised and rotationally symmetric functions *S_lw_*(*θ*), *S_sw_*(*θ*) and *P_sw_*(*θ*) are used in the following.

For reference, the projected area calculated based on our model is compared to the commonly used function *f_p_*(*θ*) [2] being used by many approaches to calculate the body surface exposed to solar radiation. Since this projection factor *f_p_*(*θ*) is solely used to calculate the impact of direct solar radiation, it is defined for the upper hemisphere (*θ* > 0°) only, whereas our function is defined on the full sphere.

**Figure 3:**
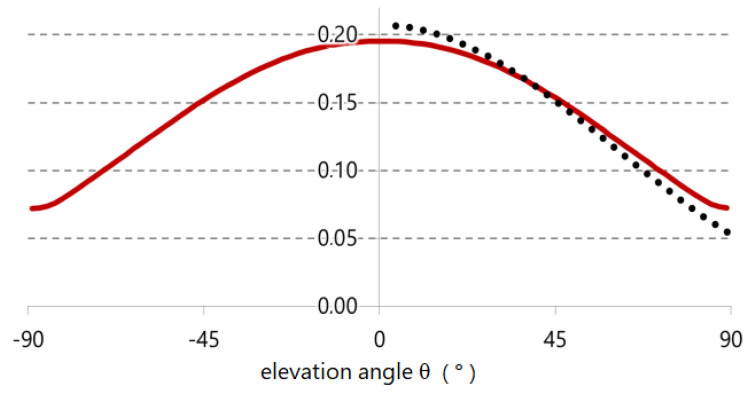
Comparison of the projection factor/angular shortwave radiation sensitivity 1/*A_eff_*_,*lw*_*·P_sw_*(*θ*) (red), determined here, vs. *α_eff_*/*ε_eff_*·*f_p_* according to Fanger (dotted black) (unitless quantity).

For this comparison, the Fanger function was weighted corresponding to our human dummy model using *α_eff_* = 0.591 and *ε_eff_* = 0.877 as surface-weighted mean values. A good match of the two functions can be seen (see Figure 3). The variability of the sensitivity function determined here is slightly lower.

### 2.3. Detection of Radiant Exitance Using UAVs

In our approach, cameras mounted on UAVs (drones) were used to determine the emitted thermal radiation (longwave), as well as the reflected global radiation (shortwave) by each surface. For this purpose, the drones have to be equipped with a thermography camera, as well as with a multispectral (or ideally hyperspectral) camera. Since we evaluated thermal comfort for public spaces and at pedestrian levels only, ground-based imaging surveys could also be considered. Nevertheless, using drones for this task has proven to be far more efficient, and the additional data acquired (e.g., roof temperatures, temperatures in courtyards) can be used for supplemental microclimatic investigations. However, the decisive advantage is the remarkably short time required for the detection flights. During the short survey period, environmental conditions will usually not vary significantly, allowing the capture of a quasistatic thermal environment and the negligence of any dynamic effects.

### 2.4. Assumption of Lambertian Emitters

For the measurement of the radiant exitance, as well as for the later sampling of mean radiation temperatures, ideal, diffuse optical surface properties following Lambert’s law are assumed. In this case, the radiance towards an observer is independent of the viewing direction. This assumption allows a significant simplification of the calculation process. In the ideal case of a Lambertian surface, the radiant intensity caused by the emission or reflection can be expressed as $I\left(\theta \right)=I_{0}\;\cdot \;cos\theta$. Hence, radiance L turns into a constant (i.e., directionally independent) value because as *L =* dI(θ)/(dA· cosθ), the cosθ term cancels out. Consequently, the radiant exitance M of the emitting surface stands in a constant ratio to L. Integration over the hemisphere shows that M=L·π. In the following, we therefore mostly refer to the radiant exitances *E_lw_* and *E_sw_* of the surfaces, albeit, practically, the radiance *L* is relevant for both the detection, as well as the sampling process. The factor π to convert between them is applied where necessary.

### 2.5. Detection of Longwave Radiation (Thermal Radiation)

It is generally assumed that the intensity values recorded by the pixels of a fully radiometric infrared camera correspond directly to the radiant exitance of the surface depicted. This is only valid if two (plausible assumptions) are made: Lambertian surface properties (see above) and grey body emission. The latter means that the emissivity is independent of wavelength, so that the measured radiation in the range of the detector (often 7.5–14 µm) stands in a constant ratio to the entire emitted thermal radiation. The grey body approximation is commonly used, and cameras are calibrated based on this assumption. Beyond the relevance in the detection process, the two assumptions made (Lambertian/grey body) are also required to allow essential simplifications in the following calculation process. Fortunately, the assumptions are well suited for the relevant mineral or naturally rough urban surfaces.

It should be noted that for our approach, we are not interested in the actual surface temperatures, but in the intensity of the radiation that these are emitting, the physical quantity being relevant for thermal comfort and directly detected by the thermography camera. This has two important implications: firstly, we do not have to determine or define emissivity values for specific surfaces; secondly, any additional diffusely reflected thermal radiation (for surfaces with *ε* < 1) is already included in the measurement.

Since the diffuse reflection of the surface is caused by its roughness, the most significant deviation regarding the Lambertian assumption occurs for the flat surfaces of glazings. These artificial, almost perfectly flat surfaces reflect a substantial amount of short- and long-wave radiation specularly, especially at or near-grazing angles. For urban environments exhibiting a significant amount of glass surfaces, further refinement of the method taking into account specular reflections is planned. However, a potentially elaborate prerequisite for this is the identification of all glass surfaces in the city model used.

### 2.6. Processing of Reflected Shortwave Radiation (Global Radiation)

While the radiation detected in the longwave spectral range consists mainly of emitted thermal radiation with a small amount of (diffusely) reflected radiation, the second component considered (and here referred to as shortwave radiation) comprises diffusely reflected global radiation reflected from surfaces, diffuse radiation scattered from the sky and direct solar radiation. While the latter two components can be described using standard models, the diffusely reflected shortwave radiation requires similar processing as longwave radiation.

Again, we have to assume ideal, diffusely reflecting surfaces according to Lambert to simplify the calculation process, and again, this represents an acceptable approximation for the relevant urban surfaces. A more detailed model could be achieved based on the use of bidirectional reflectance distribution functions (BRDF) of the surfaces involved. However, this would increase the complexity considerably, as it would require that all surfaces be classified and assigned with suitable, specific BRDF functions. Corresponding to the considerations made for the longwave radiation, the next possible and most efficient refinement of our method would involve modelling specular reflection on glass surfaces.

For the detection of shortwave radiation, ideally, a hyperspectral camera, being able to capture most of the relevant global radiation spectral range from approximately 250 to 2500 nm, was used. However, a reasonable approximation can be attained with less expensive multispectral cameras. Ban-Weiss [19] performed airborne albedo measurements of roofs and demonstrated that useful approximations could even be achieved based on conventional RGB cameras. For certain dark roofs with weak reflection in the visible spectrum, however, using a multispectral camera, offering an additional NIR channel, improved the results significantly.

**Figure 4:**
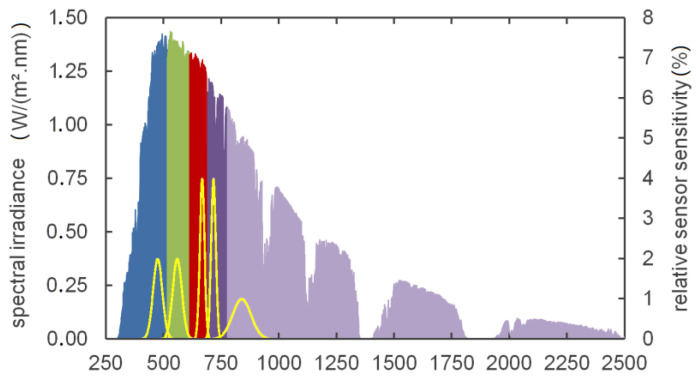
Global radiation reference spectrum segmentation and sensor functions (yellow) of the multispectral camera used (red, green, blue, red-edge, NIR).

Using a multispectral camera to determine the entire irradiance in the global radiation spectrum relies on the assumption that the limited number of narrow bands measured by the camera’s sensors can be used to approximate the total spectral irradiation of the reflected light. To do this, we developed and implemented the following approach:

Since the multispectral camera used contained two extra channels in the red to near-infrared region, the global radiation reference spectrum ASTM G-173-03 [20] is divided into five spectral segments or bands denoted as blue, green, red, red-edge, and NIR (see Figure 4). The boundaries of the segments are defined by the wavelengths in the middle of neighbouring sensor central wavelengths and the limits of the global radiation spectrum (250 nm, 2500 nm). For each of the five bands, the ideal sensor signal is determined by integration of the spectral irradiance over the sensor function defined by the central wavelength and FWHM parameters as stated in the camera’s datasheet. Each sensor signal is related to the irradiance of the entire assigned segment to determine a scaling factor for each band. The key parameters and scaling factors are listed in Table 2.

Finally, the scaling factors *C* are used to estimate the total radiant exitance *E_sw_* for each pixel using the calibrated sensor signals *s*. Using vector notation, we can express the weighted summation simply as:

(3) $$E_{sw}=\vec{C}\cdot \vec{s}=\left(\begin{array}{c} C_{blue}\\ C_{green}\\ C_{red}\\ C_{rededge}\\ C_{NIR} \end{array}\right)\;\left(\begin{array}{c} s_{blue}\\ s_{green}\\ s_{red}\\ s_{rededge}\\ s_{NIR} \end{array}\right)$$

**Figure 5:**
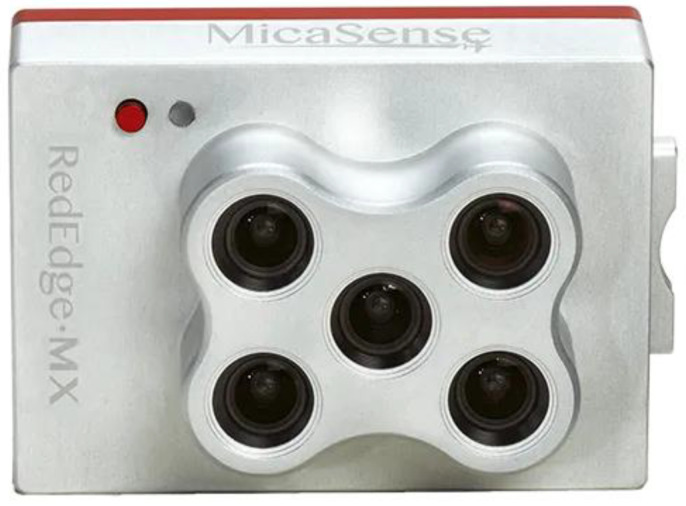
The multispectral camera used (MicaSense Red-Edge Mx).

However, before this summation for each pixel can be carried out, several essential image processing steps are necessary. Firstly, each image frame, containing only raw sensor count values, has to be converted to a matrix of radiance values, and secondly, the five spectral images have to be aligned precisely to carry out the spectral summation. The alignment, in turn, requires a prior rectification of the image. Unlike standard RGB cameras, each spectral band of the multispectral camera is detected by a separate sensor chip having its individual lens system (see Figure 5). Consequently, the processing steps have to be performed individually for each detector.

To perform the radiometric processing of each frame, the guidelines of the manufacturer were implemented. The necessary calibration parameters can be extracted from the metadata contained in the image header of each frame. The radiometric processing consists of a number of corrections based on the actual gain setting and exposure time, as well as a vignette correction. The necessary steps were well described, e.g., in Section 2.3 of Mamaghani et al. [21].

After the radiometric correction, the lens distortion has to be corrected. Again, the process has to be performed individually for each lens/sensor system, and again, the necessary distortion parameters can be found in the calibration data of the camera, saved in the header data of each image file. In the next step, the five, now rectified, separate images have to be aligned precisely, to ensure that the information in each pixel of each frame corresponds to the same surface. Since the camera can be regarded as a set of five individual cameras, there is a small, but significant variation regarding the location and orientation of each sensor/lens system. The related alignment parameters depend on the distance to the depicted objects and therefore have to be calculated for each viewing position individually. A dedicated image processing routine was developed and implemented for the project to perform the alignment of the five separate spectral images. The algorithm identifies edges in each frame to determine the optimal alignment of all frames, by maximising the edge overlap of each frame. For this purpose, a simple 2D Euclidean transformation, consisting of a translation and rotation is sufficient. Finally, a quality parameter Q is calculated to determine the plausibility of the alignment parameters found. Images with poor alignment quality are skipped. Practical application showed that only images taken from a short distance during take-off and landing could not be aligned and had to be discarded.

**Figure 6:**
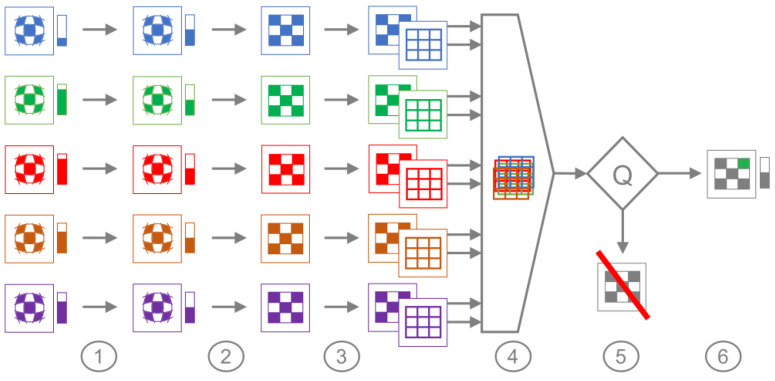
Schematic multispectral image processing.

On overview of the entire processing is depicted in Figure 6. The following steps are carried out in the multispectral processing algorithm:

1. Radiometric evaluation using camera calibration parameters (see above);
2. Individual image rectification using camera calibration parameters;
3. Individual extraction of edge features (edge detection);
4. Determination of translation vectors and rotation angles to align images based on edge features and a simple regression algorithm calculation;
5. Calculation of alignment quality parameter Q;
6. If alignment quality Q is sufficiently large: the generation of a merged image (cropping pixels containing not all five channels).

The elaborate process results are merged images with 16-bit RGB colour depth containing the approximated shortwave radiant exitance values. Since this radiometric information could be stored in a single channel (greyscale image), it is possible to store supplemental information in the RGB format. To improve the image contrast for the subsequent photogrammetric processing, as well as to facilitate the extraction of vegetation objects (see below), a simple “vegetation highlighting filter” is implemented in algorithm. While the red and blue channel of the image directly contain the radiant exitance values $E_{sw}$ (according to Equation (3)), the green channel contains an additional term defined by the ratio of NIR radiance to the blue band radiance. The radiant exitance data are therefore mapped onto the RGB values of the pixels by:

(4) $$\left(\begin{array}{c} r\\ g\\ b \end{array}\right)=\left(\begin{array}{c} E_{sw}\\ E_{sw}+3000\cdot E_{sw,NIR}/E_{sw,blue}\\ E_{sw} \end{array}\right)$$

**Figure 7:**
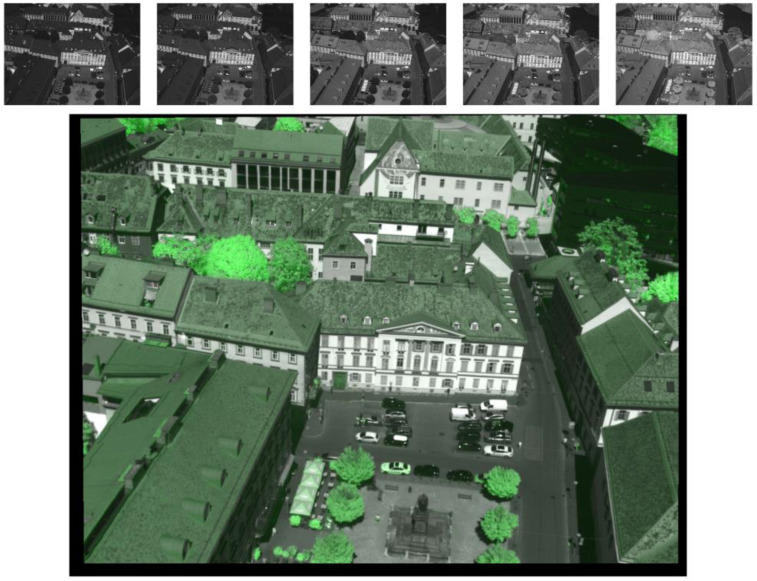
Raw multispectral images (**top**) and processed image (**bottom**) containing merged shortwave radiance data, as well as additional vegetation highlighting.

While chlorophyll absorbs strongly in the visible light range, radiation in near-infrared is largely internally scattered and reflected [22] in the leaf and on the leaf surface. Therefore, the ratio as defined for the green channel in Equation (4) is a simple, but efficient way to highlight vegetation in the multispectral images. Figure 7 shows a readily processed image containing the merged radiometric information of all five sensors, including the supplemental vegetation highlighting. The black border indicates cropped regions where information is missing due to the image alignment transformations.

The elaborate process to determine the diffuse shortwave reflections has to be considered an approximation still, since the actual measurement was performed on a limited number of channels covering only relatively narrow bands of the entire spectrum. However, the validation measurements using vertically and horizontally mounted pyranometers proved that the method is able to produce accurate results. Our thermal comfort calculation method, therefore, represents a significant refinement compared to other methods. Most methods either omit diffuse reflection of surfaces or use a constant fraction of global horizontal radiation for this purpose. Quantitatively, in terms of the total error of the final comfort calculation, the impact of the remaining inaccuracy regarding the diffuse shortwave component is very limited.

### 2.7. Photogrammetric Processing

In this processing step, both image series (longwave and shortwave) are separately converted into two three-dimensional point clouds using widely used photogrammetric software tools. For precise alignment and rectification of the model, georeferencing is applied, i.e., a few characteristic points of the model are related to geodetic coordinates. The computationally expensive calculations can be carried out using standard photogrammetric tools. The methods rely on images showing details with sufficient contrast. For the thermographic images, the pixel values reflect the brightness temperatures of the surfaces depicted, i.e., the corresponding black-body radiance temperature. This implies that geometrically relevant points, such as corners or edges, can show very little to no contrast if surface temperatures do not vary locally, making the photogrammetric process considerably more demanding. In order to overcome these difficulties, a thermographic camera offering high resolution, thorough flight planning, as well as experience in handling photogrammetric processes are required.

In a second step, the point cloud containing the multispectral data is used to compute a triangular mesh. Again, this can be carried out using commonly used software tools. This step is necessary because vegetation objects such as trees or bushes are not at all, or not sufficiently accurately, represented in the 3D city model. These vegetation objects are therefore manually cut out of the created triangular mesh and appended to the city model as an extra layer. The supplemental vegetation highlighting (as described above) helps to make this manual processing more efficient and potentially allows automated processing in the future.

In an alternative approach, the entire generated mesh could be used instead of the pre-existing city model for further processing. The advantage of this approach would be that the generated mesh represents a very accurate and current state of the region analysed. However, using a pre-existing 3D city model offers several other advantages: greater clarity of the mesh (fewer subsurfaces and noise), semantic information (classification of the surfaces, e.g., roof, facade, road, etc.), as well as potential interfaces to other formats and tools (e.g., CityGML, GIS).

### 2.8. Mapping on the 3D Model

**Figure 8:**
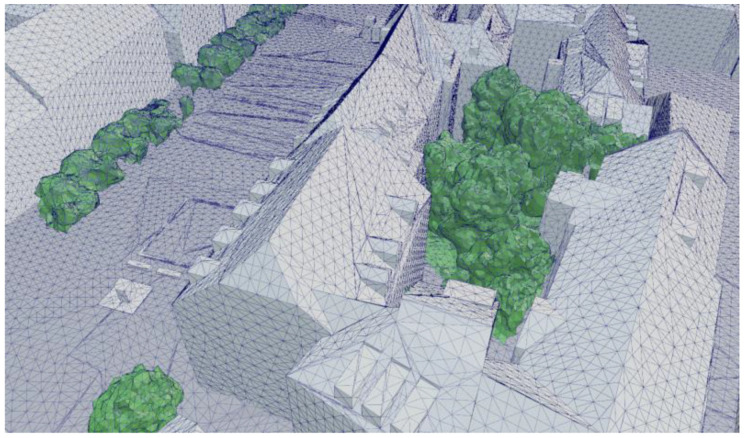
Detail of the automatically refined city model (grey) with the added vegetation layer (green).

In this processing step, the two point clouds, containing long- and short-wave radiance information, are mapped on the city model, which now includes the added vegetation layer. For this purpose, a new algorithm was designed and implemented as a software tool. Each of the two point clouds is processed separately, but the same method is applied. Before the actual mapping process, the city model mesh surfaces are automatically refined to meet a defined dimension criterion. The longest edges of the subsurfaces are split until the lengths of all edges, as well as the area of each subsurface triangle fall below a predefined value (see Figure 8). In the presented case, maximum edge lengths of 2 m and a maximum area of 1 m^2^ were applied.

After the mesh refinement, a location fine alignment of the 3D model vs. the point clouds (obtained as described in Section 2.7) is carried out. Next, the point data are mapped onto subsurfaces based on a proximity criterion. The criterion is defined based on suitable threshold values and by using either the normal distance or the minimum distance depending on the size and shape of the subsurface.

Finally, the intensity values of the points assigned to each subsurface are used to describe the radiant exitance of the subsurface. If only a small number of points is mapped onto a subsurface, a simple arithmetic averaging of the pixel values is performed. If a larger number of pixels is available, bilinear interpolation is applied to calculate a gradient for increased accuracy.

After the entire mapping process is completed, the radiant exitance in the long- and short-wave range is available in the form of attributes for each subsurface, either as a constant value or as a 2D function with a specific gradient orientation.

All processing steps regarding the import of city model and mesh refinement, as well as the point cloud mapping process were performed based on a dedicated programming library [23] implemented as pascal code. Although today, not very widespread, this programming language is compiled into native machine code and therefore allows very fast execution.

### 2.9. Model for Direct Solar and Diffuse Sky Radiation

The process described above covers only the (reflected and emitted) radiant exitance of building, ground and vegetation surfaces. Direct solar radiation and (short-, as well as long-wave) diffusely scattered radiation from the sky are described using common models. The reason for that is twofold: on the one hand, the assumptions made to detect the radiation of surfaces are only partially fulfilled by the atmosphere; on the other hand, simple and sufficiently accurate models are available for this purpose and allow simple parameter sensitivity studies. Atmospheric downward radiation $\;E_{sky,lw}$ is modelled using the Ångström equation (see, e.g., VDI, 1994 [24]):

(5) $$\;E_{sky,lw}=\sigma \cdot T^{4}\cdot \left(0.82-0.25\cdot 10^{-0.0945\;p}\right)\cdot \left[1+0.21{\left(\frac{N}{8}\right)}^{2.5}\right]$$

where *T* represents the near-surface air temperature, *p* is the water vapor pressure, whereas *N* stands for the cloud cover in oktas (0–8).

Shortwave diffuse and direct solar radiation are also described based on simple standard models. The diffuse radiation is assumed to be isotropic, and the separation of global radiation into direct (*I_dir_*) and diffuse (*E_sky,sw_*) components is performed based on the local pyranometer measurements, solar position and the Reindl decomposition model [25]. The precise relative position of the Sun is calculated using local time and geographic coordinates based on the SG2 algorithm [26].

### 2.10. Sampling of Local Mean Radiant Temperatures

The aim of our method is to determine the mean radiant temperature *MRT* as accurately as possible. Common methods for the calculation of outdoor thermal comfort only take into account human topology for the calculation of the impact of direct solar radiation. For this purpose, the function *fp*(*θ*) depending on the solar zenith angle is widely used [2]. It represents the projected area of a human body model in the direction of the direct solar radiation. Some approaches [27,28] apply additional integrals over *fp*(*θ*) to consider global radiation reflected from the ground, from simple geometric objects or to consider diffuse sky radiation. We formulated a refined and generalised approach by taking into account the anisotropic human topology, on the one hand, as well as the detailed radiative environment, on the other hand. In order to differentiate from conventional approaches, we use the notation *sMRT* (sampled mean radiant temperature) for the mean radiant temperature calculated based on our approach.

Again, a Monte Carlo method (MC) is applied to determine the two diffuse components of the radiation temperature efficiently. MC methods are well suited to solve multidimensional integrals efficiently, and the central limit theorem further allows calculating the accuracy of the sampling results. Hence, the number of raytracing operations required to achieve the desired accuracy can be minimised. The normalised sensitivity functions *S_lw_*(*θ*) and *S_sw_*(*θ*) derived from the human model are used as the probability distribution functions (PDF) to generate a set of sampling directions for the long- and short-wave sampling process (*X_lw,i_*, *X_sw,i_*). While the sensitivity functions are used to determine the elevation angles *θ* of the sampling directions, the azimuthal angles *φ*, describing the compass direction, are distributed equally. Due to the MC sampling approach, the sensitivity functions *S* cannot explicitly be found in Equation (6), but are implicitly included in the generation of the sampling directions *X*.

Based on our approach, we can formulate the mean radiant temperature *sMRT* as:

(6) sMRT=[1σ (1N∑i=1NElw(Xlw,i)+Aeff,swAeff,lw1N∑i=1NEsw(Ysw,i)+δshadow1Aeff,lwPsw(θsun)Idir)]14

The first two terms represent the diffuse components evaluated by the use of MC sampling. The evaluation can be described as a backward raytracing method and was performed using a dedicated and tested programming library [23]. The algorithm considers the 3D city model containing the radiation data, the sky and solar model as described, as well as the anisotropic radiation sensitivity functions derived from the human model. In the backward raytracing algorithm, a large set of sampling directions *X* originating from the point of interest at pedestrian height (1.1 m) were used to calculate the diffuse irradiance components accurately. The raytracing algorithm currently does not include specular reflections. Diffusely reflected components were implicitly taken into account as—under the assumption of Lambertian surface properties—they were included in the radiance measured.

Since the method allows the application of specific absorption coefficients (*α_i_*, *ε_i_*) throughout the surface of the human, the commonly used coefficient formed by the ratio *α/ε* is generalised to *A_eff,sw_/A_eff,lw_*. Alternatively and equivalently, the ratio of the area-weighted averages “effective emissivity” and “effective absorption coefficient” could be used. This coefficient accounts for the additional longwave emission resulting from the shortwave irradiance in the (here relevant) state of radiative equilibrium, and it is therefore also referred to as the scaling coefficient.

The third term of Equation (6) accounts for direct solar radiation. The function *δ_Shadow_* represents the local shading state. We considered only binary values for this function: 1 means that the location is exposed to direct sunlight, and 0 means the direction to the Sun is obstructed. The value is determined by casting a single raytracing sample in the direction of the Sun. For the direct solar term, the scaling coefficient is simplified to 1/*A_eff,lw_* as the weighting function *P_SW_*(*θ*) already implicitly contains the effective projected area for shortwave radiation.

**Figure 9:**
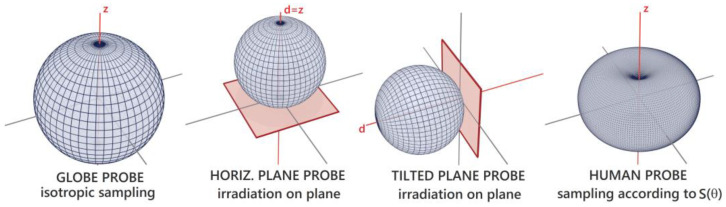
Polar plots of sampling PDFs for all probes used in the model.

For validation purposes, additional sampling models based on different angular distributions were implemented. We further refer to these sampling models based on specific distributions as “probes”, as they can be used to measure different physical quantities within the radiation model. Sampling irradiance based on the derived sensitivity functions *S*, as described above, is referred to as “human probe”. To model the measurement principle of a pyranometer, which ideally measures the irradiance on a plane, a cosine-weighted sampling was used (referred to as “plane probe”). Finally, to model the irradiance received by a globe thermometer, we applied isotropic sampling, equally distributed in all directions (“globe probe”). The PDFs for the sampling of each probe are depicted as polar plots in Figure 9.

**Figure 10:**
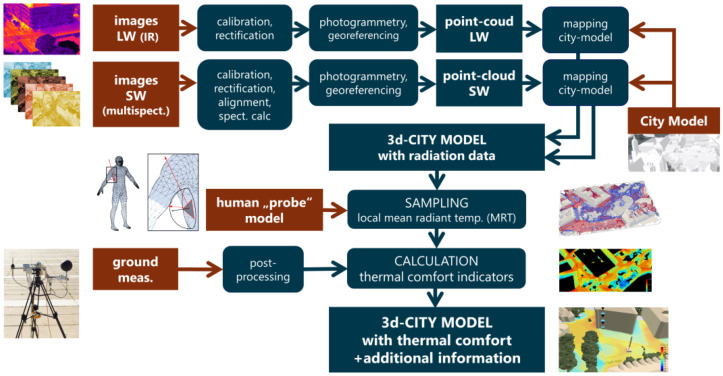
Schematic overview of the entire process chain developed.

Finally, Figure 10 gives a graphical overview of the entire processing chain of the method developed.

### 2.11. Calculation of Thermal Comfort Indices

To achieve a spatially resolved analysis of thermal comfort, sMRT was determined by performing the entire sampling procedure for each point within the area of interest. Although the sampling process was carried out in 3D, the evaluation of radiation temperatures was not carried out on a volumetric grid, but restricted to a surface at the height of 1.1 m above ground level, representing the potential locations of pedestrians. The surface was evaluated using a defined grid size (here 1 × 1 m) carrying out the entire sampling process for *sMRT* for each grid point.

The sampled mean radiation temperatures, the air temperature, the water vapour pressure, as well as the air speed are then fed into the UTCI calculation algorithm (see e.g., Błazejczyk et al., 2013 [29], and Jendritzky et al., 2012 [30]). The resulting local Universal Thermal Climate Index reflects the perceived temperature based on a commonly used and tested method to objectively measure thermal comfort or thermal stress. Furthermore, we used the same parameters to calculate thermal comfort based on the famous PMV method developed by Fanger. For this calculation method, the additional definition of a metabolic rate and the clothing factor are required. For the evaluation presented, a metabolic rate of 1.9 met (=slowly waking) and 0.26 clo (=lightly dressed) were defined.

The air body properties (temperature, humidity, wind speed) are regionally defined as constants. Spatially resolved determination and modelling of variable air body properties is a great challenge. Using few spot measurements to interpolate or extrapolate a three-dimensional field is problematic. Alternatively, precalculated field data of CFD, multiphysics or meteorological models could be imported to our calculation method. However, following our working hypothesis, compared to the spatial variation of mean radiation temperatures, the spatial variability of the air body properties has a limited effect when thermal comfort in intra-urban heat island scenarios is considered. Therefore, it is a reasonable simplification to assume zonal constant air body properties in our small-scale analysis of urban quarters. Measurements performed in our case study under midsummer conditions largely confirmed this. It should also be noted that the chilling effect of wind decreases as air temperature increases. Consequently, the significance of knowing the precise air speed reduces.

### 2.12. Process Overview

Figure 10 provides a graphic overview of the entire process chain. The red colour highlights the input data, rectangular fields the data, whereas rounded rectangles the processes.

## 3. Application Case: Results

### 3.1. Target Area, Equipment Used and Ground Measurements

In order to prove and demonstrate the practicability and validity of our approach, we applied the entire process chain to an urban quarter of Graz, Austria. The target area is located in the historic downtown and comprises two extended paved squares, as well as narrow streets and historic buildings. Throughout the area, a relatively small number of trees can be found. The sky condition was clear with no clouds.

**Figure 11:**
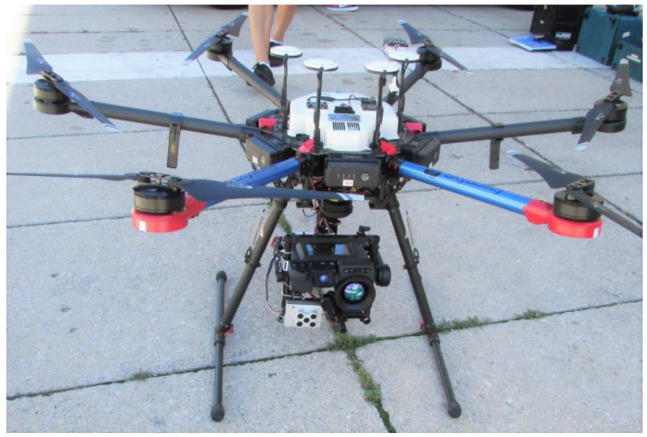
Drone Skyabilty M600 in the target area, with the IR camera (black) and multispectral camera (silver) on a gimbal system.

The drone flight and spot measurements were carried out on 20 August 2020 between 3:33 pm and 4:13 pm. For the thermographic detection, a FLIR-T1020 infrared camera was mounted on the drone. The shortwave radiation was recorded using the multispectral camera MicaSense RedEdge MX. The flight time of the drone was 10 min. During this time span, 5.3 GB of data consisting of 1200 infrared images and 1400 multispectral images were recorded by the two cameras mounted on the gimbal stabiliser of the drone (see Figure 11). Corresponding to our approach, to capture horizontal, as well as vertical urban surfaces, the drone operated at lower altitudes, only maintaining safety distances from roof top levels.

Parallel to the aerial survey, ground measurements at 12 different locations within the target area were performed. The measurements included: air temperature, wind speed, relative humidity and global horizontal irradiance, global tilted irradiance and globe temperature. These spot measurements (at a height of 1.5 m) were carried out at locations showing different solar exposure levels, ranging from sunny locations on the sealed square to well-shaded spots within the narrow streets.

**Figure 12:**
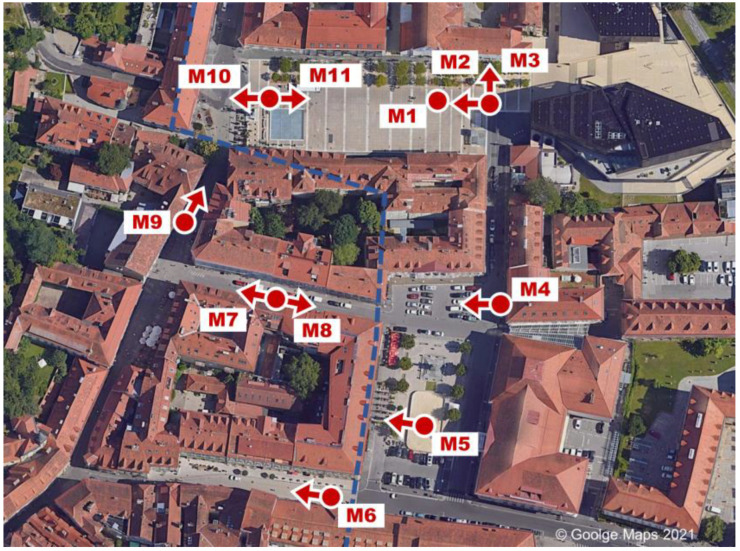
Target area with measurement spots (dots) and the orientation of the GTI measurements with a vertically mounted pyranometer (arrows).

Nevertheless, all air temperatures observations were distributed within a relatively narrow range. For the spatially resolved calculation of thermal comfort maps, the target area was split into two zones, which were assigned constant air body properties. The zone boundary is depicted as the dashed blue line in Figure 12. The average values of the air temperature measurements and wind speed were used for the evaluation. In the left Zone B, containing the narrow streets, the mean air temperature was 27.82 ± 0.28 °C, and in the right Zone A, containing the squares exposed to direct sunlight, the mean air temperature was measured as 28.98 ± 0.35 °C.

The spot measurements were carried out sequentially according to their enumeration. The measuring process for the 12 spots lasted for a period of approximately 40 min. The values measured are listed in Table 3. The mean radiant temperature (*MRT*) column included in the table was calculated according to the method specified in ISO 7726 [31].

**Figure 13:**
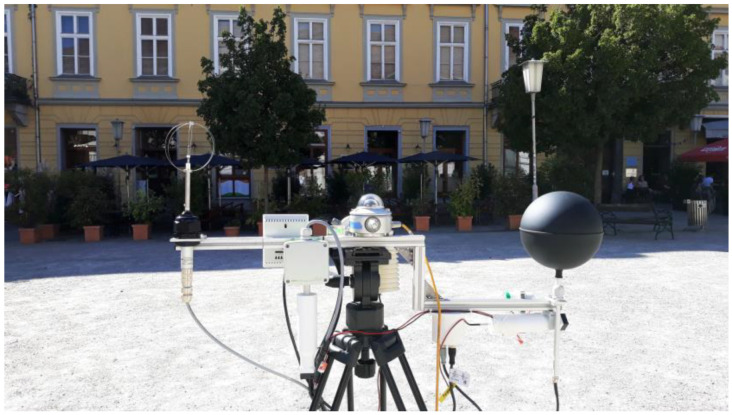
Spot measurement at location M5 (horizontally mounted pyranometer facing backwards).

Figure 13 shows the measurement equipment used mounted on a tripod. The equipment consisted of a thermo-anemometer, vertically and horizontally mounted pyranometers, a 15 cm globe thermometer, two capacitive humidity sensors and several ventilated and screened air temperature sensors (for redundancy).

### 3.2. Mapping of Radiance (Radiant Exitance) Data

**Figure 14:**
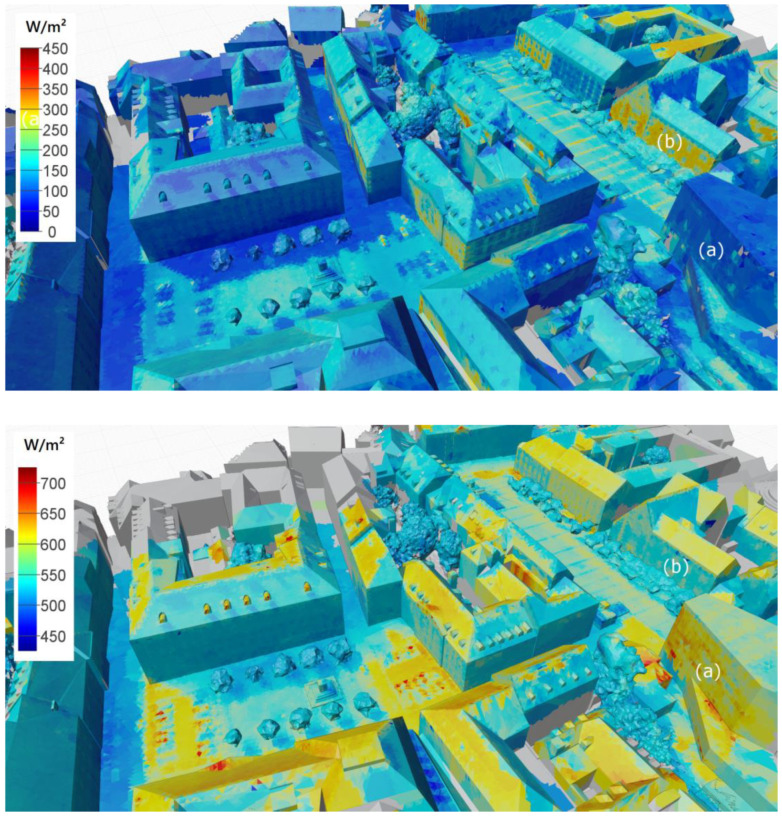
West view: radiant exitance data mapped on 3D city model (**top**: SW radiation; **bottom**: LW radiation).

**Figure 15:**
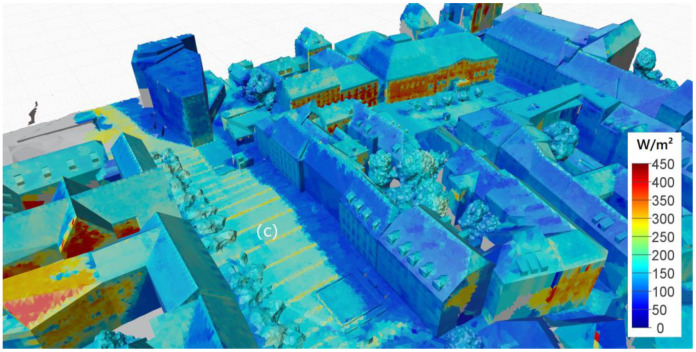

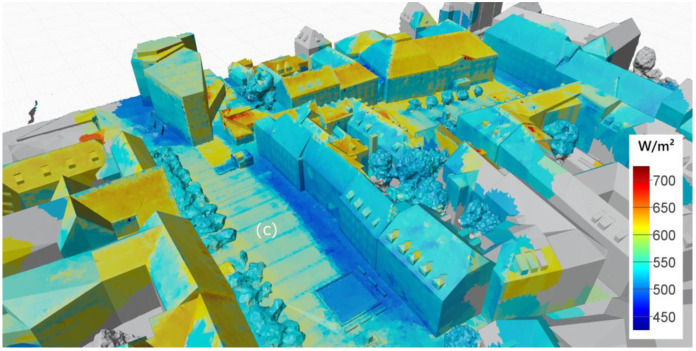
East view: radiant exitance data mapped on 3D city model (**top**: SW radiation; **bottom**: LW radiation).

Following the process described above, both point clouds, containing the radiant exitance data, were mapped on the city model. Figure 14 and Figure 15 show the processed city models containing the radiant exitance data for thermal radiation (LW) and diffusely reflected global radiation (SW). The colour mapping for each image was chosen in a way that allows comparison of the impact of the radiation on the thermal comfort sensation. In order to establish this comparability, the minimum value for the longwave scale is defined as 425 W/m^2^, equivalent to a black-body radiation temperature of approximately 21 °C. This temperature can be defined as neutral regarding the radiative exchange for most thermal comfort models. The LW scale then spans over an interval of 300 W/m^2^ to 725 W/m^2^ (equivalent to approximately 63 °C). To put the SW scale into a physiologically equivalent range, we divided the 300 W/m^2^ range of the LW by the scaling factor for our human model (*α_eff_*/*ε_eff_* = 0.67) to obtain a range from 0 W/m^2^ to 450 W/m^2^. Following this approach, surfaces showing the same colours have an equivalent impact on the thermal comfort sensation. The comparison reveals the thermal comfort impact of reflected SW and emitted LW radiation range on a comparable scale. However, while the effective LW radiation values are higher on average, the variation of the SW component shows greater variability.

Further, the complementary effect regarding the surface colour can be seen quite well. While dark surfaces reflect only a small fraction of the incoming solar radiation, their surface temperatures are generally higher (e.g., see the modern building marked as (a) in Figure 14). In contrast, bright (white) surfaces will reflect the shortwave radiation and maintain lower surface temperatures (e.g., see the historic building marked as (b) in Figure 14). The effect is also clearly visible on the pavement of the public square (see mark (c) in Figure 15), where rows of white paving stones with a width of approximately 30 cm interrupt the otherwise grey pavement.

### 3.3. Validation of Shortwave Radiant Exitance and Sampled Shortwave Irradiance

**Figure 16:**
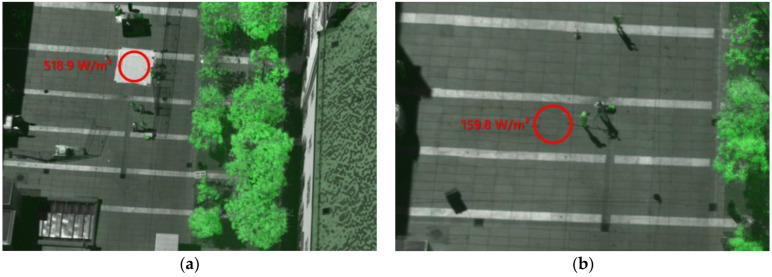
Images used for the validation of the shortwave radiant exitance detection and processing ((**a**): using a PP-fleece; (**b**): pavement).

Two approaches were taken to validate the detection and processing chain for the shortwave radiant exitance. In the first approach, ground reflection measurements with a pyranometer were compared against processed multispectral drone measurements. In the second approach, pyranometer measurements were compared against measurements in the finished processed model. For the first approach, the radiant exitance of two different surfaces was directly measured on the ground with a downward-looking pyranometer. This setup is known from albedo measurements. The pyranometer was mounted on a stick of 2 m in length at a height of 50 cm. In the first measurement at 12:49, the ground below the pyranometer was covered with a white polypropylene fleece with a dimension of 4 × 4 m to increase the albedo of the surface. In the second measurement at 15:29, the ground, paved with grey stones, was left uncovered. Both measurements were simultaneously recorded during drone flights. The images of the multispectral camera were processed as described in Section 2.6 to obtain the total radiant exitance. The results of the radiometric image processing are shown in Figure 16.

The mean values of all pixels in the range of the circular area marked in the images were used to determine the measured radiant exitance. Furthermore, the standard deviations of the pixel values were calculated. The results were validated against the measurements of the pyranometers performed at the same time. Furthermore, the data of a second, upward-looking pyranometer were used to calculate the albedo of each surface.

The results of the validation, shown in Table 4, revealed that, although numerous processing steps were involved and although the spectral resolution of the multispectral camera was limited, the resulting accuracy was surprisingly good. To further include the entire mapping and sampling process in the validation, additional measurements with a horizontally looking (=vertically mounted) pyranometer were carried out during the flight and compared to model measurements. To determine the required, but unknown values of direct solar irradiance (DNI) and diffuse sky radiation (DHI), the upward-looking pyranometers measurement data were used. The diffuse component of the horizontal pyranometer measurements in urban environment comprises diffuse radiation from the locally visible sky area, as well as reflected components of facades and vegetation. To consider the local variation of DHI values “virtual measurements” of the global horizontal irradiance (GHI) at the precise positions of the ground measurement were carried out in the model. For this purpose, the “horizontal plane probe” (see Figure 10) was used to simulate the pyranometer measurements in the model. The meteorological DNI and (unobstructed) DHI irradiance input parameters to the model were then varied to obtain the best match with real-world measurements. The match quality was measured by the mean deviation of the GHI values for real-world vs. model measurements.

**Figure 17:**
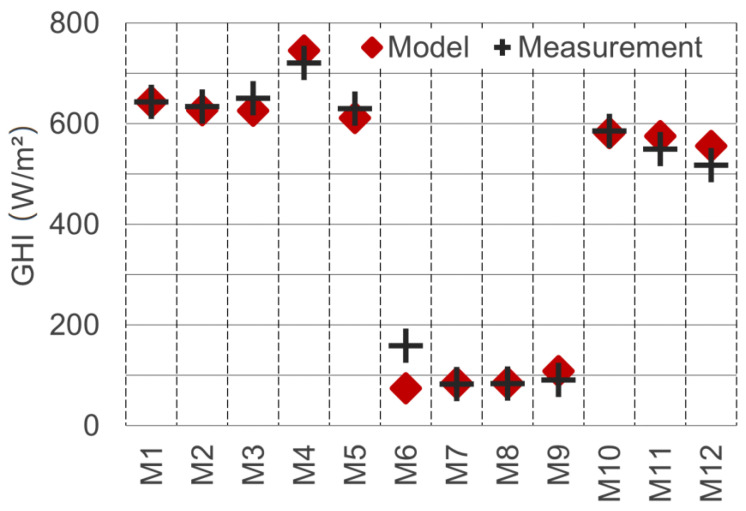
GHI values of real-world and model measurements after matching of the DNI and DHI components.

Following this approach, the global DNI and DHI parameters for the model were determined as 790 W/m^2^ and 90 W/m^2^, respectively. Using these values, a good overlap of model and measurement could be achieved (see Figure 17). The average GHI error when using these values was minimised to 20.9 W/m^2^. The resulting coefficient of determination R^2^ showed an excellent model/measurement match of 0.986. Since the model irradiance data (DNI and DHI) were calibrated based on the measurement data, the comparison of the GHI values can only partially be considered a validation. For further validation of the achieved irradiance parameters, they were compared against weather station data. Calculating the meteorological GHI (with unobstructed horizon) for the local time 15:45, we obtained 609.8 W/m^2^. The closest public weather station (“Graz-Nord”) records half-hourly mean values. The station is located at a distance of 2.7 km and recorded an average measurement value of 612.0 W/m^2^ for the period between 15:30 and 16:00. The low difference of 0.4% supports the calibration approach and confirms the method’s accuracy.

For simplification, the determined irradiance parameters DNI and DHI were further taken as constant for all evaluations performed. The precise position of the Sun, however, was calculated for each spot measurement time individually.

**Figure 18:**
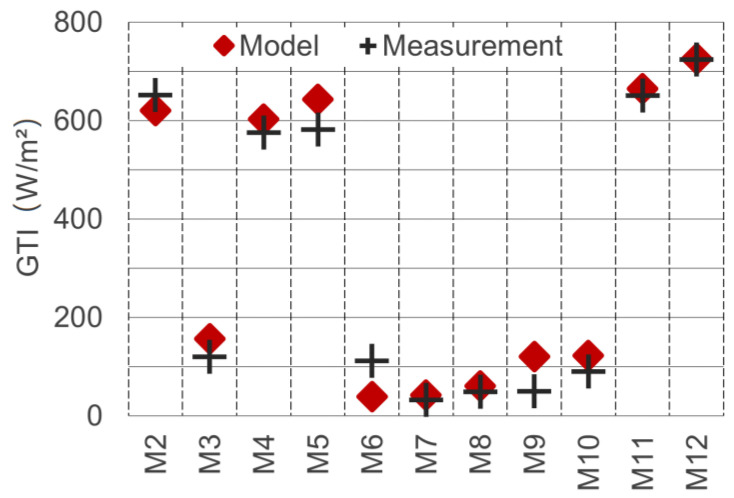
Global tilted irradiance GTI on vertically mounted pyranometer real-world vs. model measurements.

The last and most comprehensive validation step for shortwave detection was carried out by comparing real-world measurements of the vertically mounted pyranometers against the equivalent measurements in the model. Unlike the horizontal measurements, the vertical measurements were not part of the calibration process just described. In the model, the “tilted plane probe” (see Figure 10) was used to measure the total irradiance on the vertically oriented planes (here referred to as GTI, for global tilted irradiance). The results of the real-world and model measurement are depicted in Figure 18.

The measurement spot M1 is missing in the chart, because the pyranometer was used for the albedo measurement at this location. The R^2^ value for the match model/real-world for the vertically oriented measurements is 0.982. The average deviation is 33.2 W/m^2^. The deviation can, on the one hand, partly be attributed to the imperfect model geometry match, due to a limited level of detail for buildings and vegetation. On the other hand, the assumption of ideal Lambertian surfaces causes deviations regarding the detection, as well as the simulation process.

### 3.4. Validation of Longwave Exitance and Sampled Mean Radiant Temperatures

To validate the determination of the longwave radiation and mean radiant temperatures, model measurements with isotropic sampling (*globe probe* model in Figure 10) were carried out. For this purpose, both components, LW and SW irradiance, have to be considered. The mean radiant temperatures sampled in the model (*sMRT*) were compared to the real-world ground measurements performed.

The empirical determination of the mean radiant temperature in summerly outdoor environments is a challenging task. We faced three main issues regarding the measurements with the globe thermometer:

1. Especially for high MRT values (exposure to direct solar radiation), the MRT calculation of measured globe temperatures according to the ISO 7726 depends strongly on the measured wind speed. In hot urban environments, convective air movements are very unstable and fluctuate in strength, as well as direction. A model including such effects considering dynamic effects and turbidity was not available and would require additional measurements;
2. Especially for exposure of the globe thermometer to direct sunlight, the shortwave absorption coefficient *α* has to be known precisely to model the behaviour of the globe thermometer. Unfortunately, even upon inquiry, the manufacturer of the globe thermometer (Swema AB) could not provide any information regarding the shortwave absorption of the globe’s coating;
3. The thermal inertia of the globe thermometer would require an extended period of time to reach thermal equilibrium with the environment, particularly when changing from a Sun-exposed to a shaded location.

**Figure 19:**
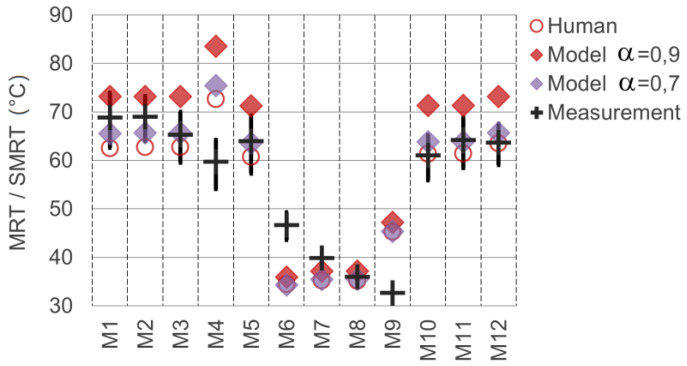
Comparison of mean radiant temperatures measured in real-world vs. sampled in the model (data with human sampling for reference).

To address the first issue, we used time-averaged values of the actual measurement period to determine the wind speed. For reference, however, we also provide two additional MRT values, the calculated wind speed 0.5 m/s higher and lower than the measured average value. These cases reflect the upper and lower edge of the black cross markers depicted in Figure 19. The evaluation of errors and R^2^ values was performed using the mean value only.

Regarding the second issue, namely the absorption coefficient of the MRT globe, the markedly visible specular reflection on the globe surface (see Figure 13) already shows that a value significantly lower than 1.0 has to be assumed. Furthermore, it has to be considered that specular reflection on the comparably flat surface increases considerably with the angle of incidence (theoretically reaching 100% for grazing incidence on any surface). Since the average incidence angle of the direct solar beam on the spherical surface of the globe thermometers is 60°, the effect of specular reflection would be significant, reducing the effective absorption. Thus, we performed two model measurements using different shortwave absorption coefficients *α* of 0.7 and 0.9 (equal to albedo 0.3 and 0.1). To mitigate the impact of the third issue, we clustered the measurement locations regarding their exposure to direct sunlight (with locations M6 to M9 being shaded). Still, the effect of thermal inertia could not be entirely compensated. While the individual measurements would require longer periods, the total duration of all measurements should be as short as possible to capture a quasistatic environment.

Table 5 shows the results of the MRT measurements and evaluation with the timely mean values, as well as the values for reduced or increased wind speeds. The columns on the right side show the model measurement results using the isotropic sampling (“globe-probe”) for two different assumed absorption coefficients. The last column is for reference only and shows the sampling results at the measurement locations when using the “human probe” model.

At the bottom of the table, the mean errors and the coefficients of determination R^2^ are listed. The measurement locations M4, M6 and M9 show significant deviations. The deviation of M6 can clearly be assigned to the thermal inertia effect since this was the first measurement taken in the shadow and the measurement period was not sufficiently long to reach thermal equilibrium. A smaller inertia effect might still contribute to the measurement deviation of M7. Furthermore, the downward deviation of M10, which reflects the first location exposed to direct sunlight again, is likely caused by thermal inertia.

The significant outliers M4 and M9, however, are caused by variations of the mapped radiation data in the model. Both locations are located very close to facade surfaces (see Figure 12) and are therefore strongly affected by local fluctuations of the radiant exitance in the nearest subsurfaces of the model. Additionally, M4 is located in front of a parking space, and reflections of the cars might have led to deviations in the model. In general, locations at “open” squares (M1—M3, M5, M10—M12) show less deviation since any mapping fluctuations will quickly be averaged out as soon as a certain minimum distance to the objects is exceeded. Following these considerations, a second evaluation of the results with the three most significant systematic outliers removed is shown in the last two rows of Table 5. The removal of outliers is debatable of course. By removing the three outliers, R^2^ reaches a value of 0.97 for *α* = 0.7, representing a good model to measurement match.

### 3.5. Calculation of MRT and Thermal Comfort Indicators at the Pedestrian Level

Finally, the spatially resolved calculation of comfort indicators at the pedestrian level was performed. At the height of 1.1 m above the ground level, the thermal comfort indicators UTCI and PMV were calculated. For this purpose, the local LW and SW irradiation conditions were precisely sampled for each point individually. The calculation for the entire target area (40,000 m^2^) with a resolution of 1 × 1 m lasted approximately 30 h on a single standard CPU. Apart from the thermal comfort indices, the underlying spatially resolved calculation components are a valuable source of information for further in-depth analysis. Due to the nature of the approach, these parameters and any derivatives, e.g., long- and short-wave radiation temperatures, effective view factors, the composition of radiative components and radiative asymmetries can be presented as maps. For any specifically chosen location, further analysis of the thermal environment, relevant heat sources and optimisation potentials is possible by analysing local parameter dependencies.

Further, it is possible to perform simple parameter variations to study the effect of potential optimisation measures. A full elaboration of the range of potential applications is beyond the scope of this publication. The parameters currently exported as data maps are listed in Table 6.

**Figure 20:**
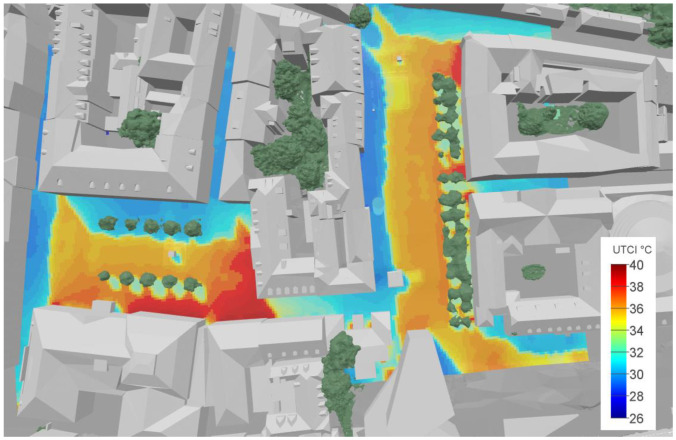
Map of UTCI indicators (“perceived temperatures”) calculated with a resolution of 1 m at a surface located 1.1 m above the ground level (pedestrians).

Figure 20 shows the calculated UTCI map in 3D view. All other result maps as listed in Table 6 can be found in Appendix A. The perceived temperature in the form of the UTCI shows a high spatial variation throughout the analysed area. The local comfort values range between 28 °C and 41 °C. The influence of the direct sunlight (shadow casting) can of course be seen very well. However, there is also a significant variation that is caused by differences in the temperatures and shortwave reflectivity of facades and pavements.

We further analysed the correlation between the calculated PMV and UTCI value in the entire target area. Since in the present case, the local variation of the thermal comfort indices is mostly affected by the variation of the mean radiant temperature, the two calculated thermal comfort measures show a very high correlation of R^2^ = 0.997. The high correlation between these two indices in outdoor urban environment was also shown by Blazejczyk et al. [32] in a comparative study.

**Figure 21:**
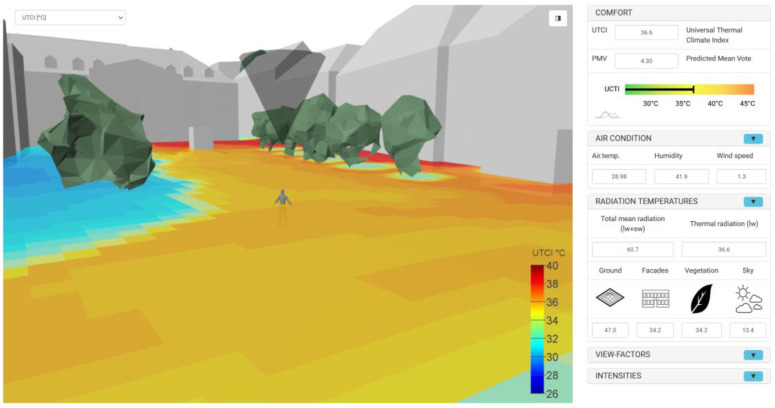
Interactive 3D web application to explore all computed information.

To provide appropriate access to the computed, extensive and multidimensional data, a proprietary, three-dimensional interactive web viewer based on WebGL technology was developed and implemented (see Figure 21). It allows viewing of the 3D city model with the mapped LW and SW radiant exitance, viewing of data layers as maps, as well as in-depth analysis for any evaluated point within the target area.

**Figure 22:**
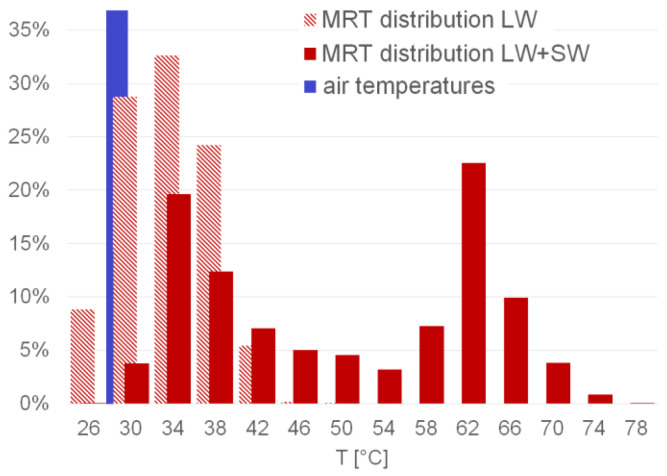
Distribution of measured air temperatures (blue) and determined mean radiant temperatures in the target area (light red: based on longwave radiation; red: based on long- and short-wave radiation).

To finally illustrate the validity of our working hypothesis, which assumes that mean radiant temperature is the key influencing parameter on thermal comfort when analysing overheating in urban environments, Figure 22 shows the distribution of all sampled mean radiant temperatures at the pedestrian level, as well as of air temperatures measured in the target area.

As can be seen in Figure 22, the measured air temperatures are distributed within a narrow range from 27.6 °C to 29.6 °C, while the mean radiance temperatures resulting from thermal radiation cover a wide range up to approximately 50 °C. If the impact of shortwave radiation is also considered, the distribution extends further, reaching maximum temperatures of up to 80 °C.

## 4. Conclusions

Based on the case study in the historic downtown of Graz, we could prove that the method proposed in this publication is practicable and generates interesting and extensive results already upon its first applications. The working hypothesis, assuming that mean radiant temperature (MRT) is the key parameter for the analysis of intra-urban heat islands, could be confirmed. This justifies the elaborate process of detecting and evaluating all long- and short-wave radiation components, as presented in our method.

The derivation of the directional sensitivity to radiation based on our human dummy revealed that standing or walking humans are most sensitive to radiation arriving at mainly horizontal angles (see Figure 2 and Figure 9). This, in turn, confirms the necessity for a detailed and three-dimensional analysis as proposed here, since radiation of these directions is reflected and emitted by vertical surfaces (such as facades or vegetation), which are often dominant in urban environments.

The detection of long- and short-wave radiation by means of cameras mounted on a UAV turned out to be very efficient. Although the radiometric measurements could also be performed from the ground, the significantly less obstructed airborne viewing positions and the short duration required proved to be beneficial to the method.

In order to accurately determine diffusely reflected global radiation, an elaborate processing chain was developed, implemented and tested. In this process, the information of five individual spectral images was calibrated, rectified, aligned and merged to derive an approximation for the total shortwave radiant exitance of urban surfaces. The validations against ground measurements with upward, downward and horizontally facing pyranometers proved the accuracy of the developed method (see Section 3.3 and Figure 18).

**Figure A1:**
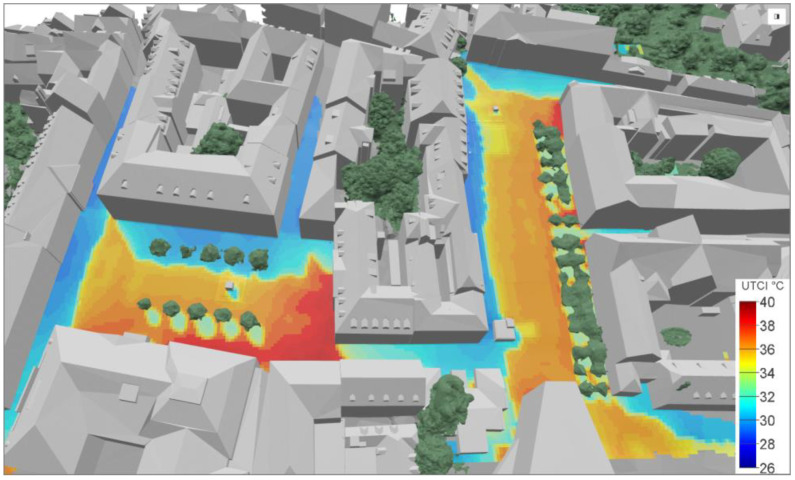
Spatially resolved evaluation of Universal Thermal Comfort Index (UTCI).

**Figure A2:**
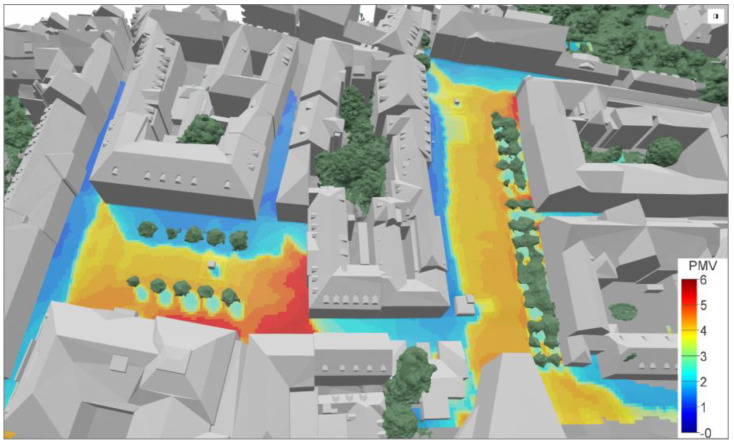
Spatially resolved evaluation of predicted mean votes (PMV) (Fanger).

**Figure A3:**
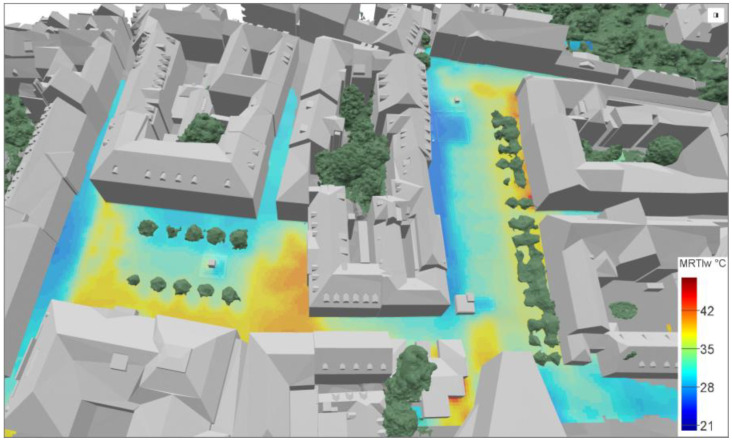
Spatially resolved evaluation of sampled mean radiant temperatures—longwave components only (sMRT-LW).

**Figure A4:**
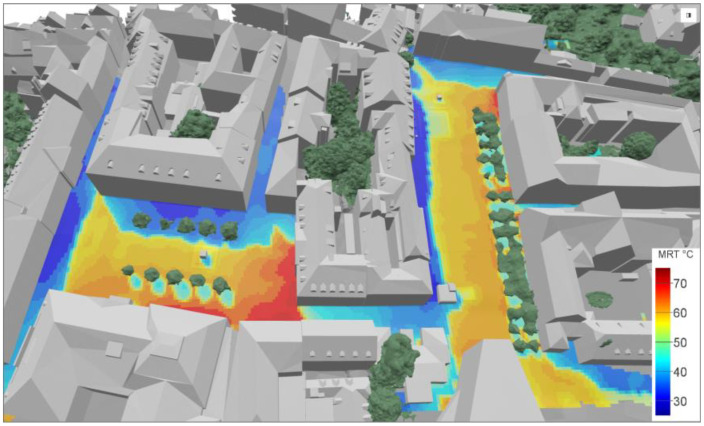
Spatially resolved evaluation of sMRT, all components.

**Figure A5:**
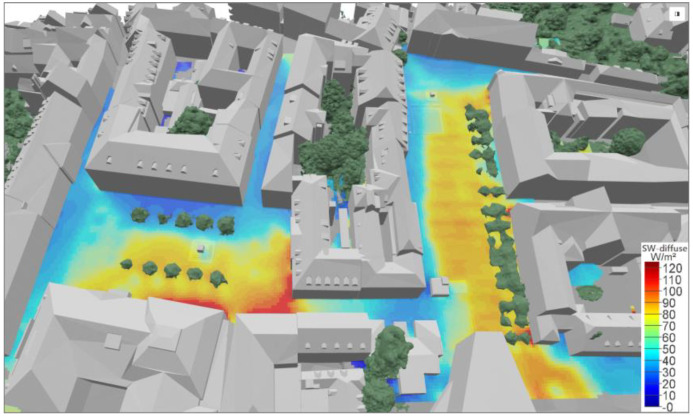
Spatially resolved evaluation of sampled diffuse shortwave components (SW-diff) (scattered from sky and reflected from facades, ground, vegetation).

**Figure A6:**
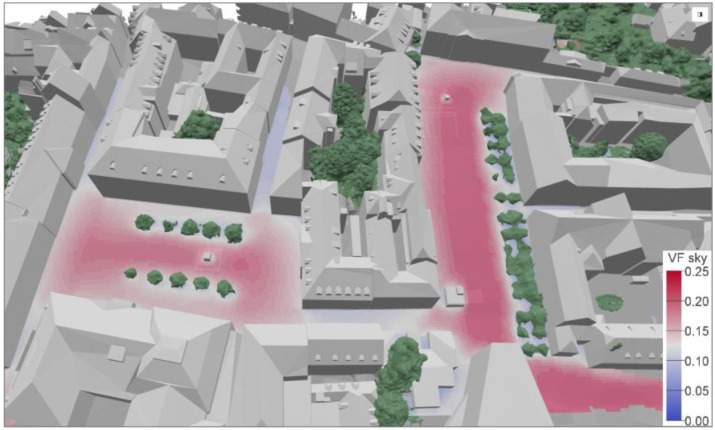
Spatially resolved evaluation of effective (physiologically-weighted) sky view factor (VF sky).

**Figure A7:**
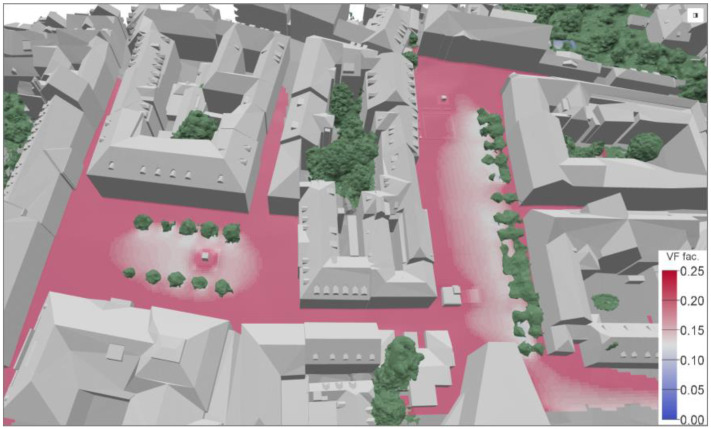
Spatially resolved evaluation of effective (physiologically-weighted) facade view factor (VF facade).

**Figure A8:**
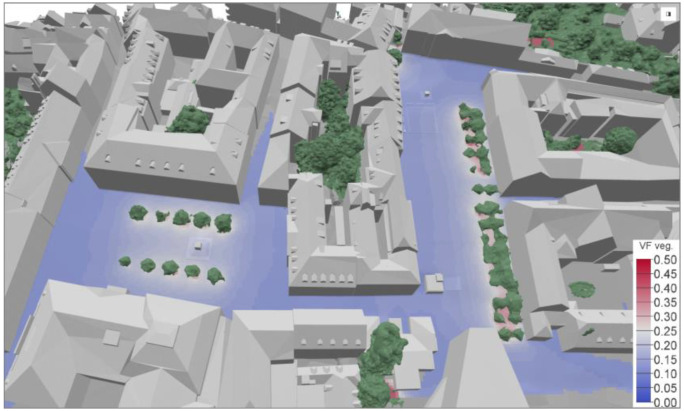
Spatially resolved evaluation of effective (physiologically-weighted) vegetation view factor (VF vegetation).

**Figure A9:**
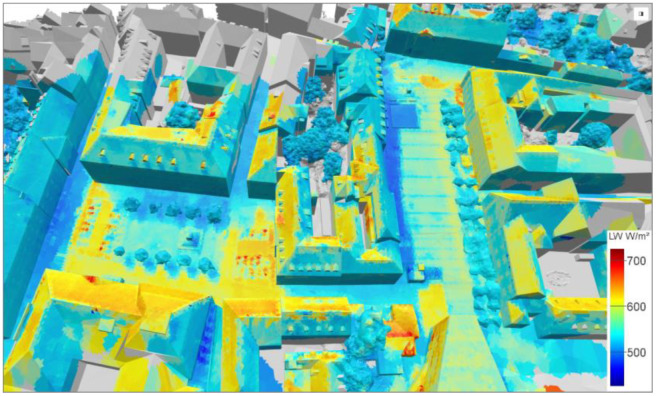
Longwave radiant exitance mapped on the city model (emitted and reflected thermal radiation).

**Figure A10:**
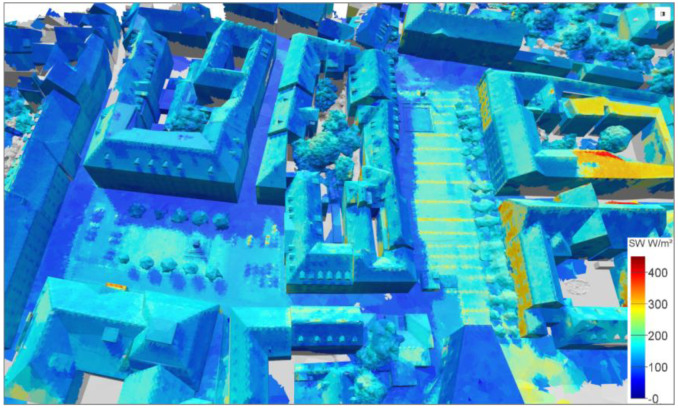
Shortwave radiant exitance mapped on the city model (reflected global radiation).

Although relying on computationally expensive and complex numerical algorithms, the method proved to work efficiently and robustly, generating plausible and spatially well-differentiated results (see Appendix A, Figure 20 and Figure A1, Figure A2, Figure A3, Figure A4, Figure A5, Figure A6, Figure A7, Figure A8, Figure A9 and Figure A10). The comparison of the UTCI maps and PMV maps calculated based on our approach shows a very high correlation of R^2^ = 0.997 (see Figure A1 and Figure A2), because the variation of MRT dominated the calculation results. The calculated UTCI values, being a measure for perceived temperatures, show a range of 28 °C to 41 °C in the area analysed. In an earlier study [14,15], being the first application of the method, the beneficial effect of vegetation (in the form of trees and grass surfaces) could be shown very well. Due to the local conditions, comprising only small trees and no grass surfaces, this effect is less visible in the present case, but can still be seen in a drop in the UTCI values near and between the trees. This effect is caused by the surface temperatures of vegetation, which are significantly lower than those of the built environment. Due to evaporation, leaf surface temperatures of healthy plants are usually even slightly lower than the air temperature. Consequently, mean radiant temperatures are significantly lower if vegetational surfaces comprise a high proportion of the visible environment.

To validate the detection, as well as the processing of longwave (=thermal) radiation, model measurements and real-world measurements of mean radiant temperatures with a globe thermometer were compared (see Section 3.4 and Figure 19). Considering the challenges of MRT measurements in such environments, the validation results are satisfying. Future validation measurements should be carried out with a set of stationary globe thermometers with a well-known shortwave absorption behaviour to achieve improved validation results.

Further development of the method will involve additional automation of the processing steps required, as well as the integration of a model for specular reflection on glazed surfaces.

We think the developed method can provide valuable new insights regarding key influencing factors and main sensitivities to assist urban planners in tackling intra-urban heat islands. The data generated with our method can further serve as a valuable source to validate and enhance microclimatic simulation models and tools, as we are able to generate spatially highly resolved results based on measurement data.

## Figures and Tables

**Table 1:** Emissivities and absorption coefficients used.

Parameter	Skin	Clothing
*ε* (lw)	0.97	0.80
*α* (sw)	0.70	0.50

**Table 2:** Spectral band segment data and calculated scaling factors.

	Spectral Segment				
	Blue	Green	Red	Red Edge	NIR
wavelength start (nm)	250.0	517.5	614.0	692.5	778.5
wavelength end (nm)	517.5	614.0	692.5	778.5	2500.0
irradiance (W/m^2^)	169.6	129.7	97.9	89.9	413.0
spectral share	18.8%	14.4%	10.9%	10.0%	45.9%
sensor signal *s* (W/m^2^)	1.302	1.344	1.256	1.077	0.883
scaling factor *C*	130.3	96.5	78.0	83.5	467.7

**Table 3:** Ground measurement results.

Name	Zone	Start Time	GHI (W/m^2^)	GTI (W/m^2^)	GTI DIR (°)	T_air_ (°C)	T_globe_ (°C)	MRT (°C)	RH (%)	v_air_ (m/s)
M1	A	15:33:00	643.7	163.1	down-wards	28.79	42.43	68.88	40.5	1.3
M2	A	15:39:00	652.2	634.8	270	29.57	41.25	69.00	41.0	1.6
M3	A	15:40:30	120.4	651.1	0	29.15	41.15	65.28	40.7	1.3
M4	A	15:42:50	576.1	720.6	270	29.18	40.00	59.75	39.4	1.1
M5	A	15:44:50	582.4	630.4	260	28.93	41.56	64.00	39.7	1.1
M6	B	15:47:10	111.8	159.3	260	28.22	33.77	46.70	42.8	1.3
M7	B	15:52:10	32.3	82.9	259.5	27.78	30.87	39.87	44.0	1.8
M8	B	15:54:10	48.5	83.9	79.5	27.58	30.05	35.96	44.3	1.1
M9	B	15:56:10	50.0	90.8	150	27.70	29.91	32.69	44.7	0.4
M10	A	16:02:40	90.6	586.1	90	28.56	40.19	61.02	41.3	1.2
M11	A	16:06:10	650.5	549.3	270	29.09	41.39	64.20	41.5	1.2
M12	A	16:13:10	724.1	517.7	270	28.55	39.53	63.64	43.1	1.5

**Table 4:** Validation of shortwave radiant exitance.

Case	Radiant Exitance M (W/m^2^) IMAGING	Radiant Exitance M (W/m^2^) PYRANOMETER	Relative Deviation	Irradiance (W/m^2^)	Albedo
PP-fleece	518.9 ± 19.2	519.3 ± 4.8	−0.1%	677.8	0.77
Pavement	159.8 ± 7.6	163.1 ± 1.6	−2.0%	643.7	0.25

**Table 5:** Validation of longwave measurements by (sampled) mean radiant temperatures.

Real-World Measurement							Model Measurement		
Spot	Start Time	Wind (m/s)	T_globe_ (°C)	MRT (°C)	MRT_v−0.5_ (°C)	MRT_v+0.5_ (°C)	sMRT_globe_ (°C)	sMRT_globe_ α (°C)	sMRT_human_ (°C)
M1	15:33:00	1.26	42.4	68.9	62.6	74.0	65.5	73.2	62.5
M2	15:39:00	1.60	41.2	69.0	63.9	73.3	65.7	73.2	62.8
M3	15:40:30	1.27	41.2	65.3	59.5	70.0	65.7	73.2	62.8
M4	15:42:50	1.10	40.0	59.8	54.1	64.2	75.4	83.6	72.6
M5	15:44:50	1.05	41.6	64.0	57.3	69.2	63.6	71.2	60.8
M6	15:47:10	1.31	33.8	46.7	43.6	49.3	34.3	35.9	34.4
M7	15:52:10	1.81	30.9	39.9	38.3	41.2	35.5	37.1	35.2
M8	15:54:10	1.13	30.1	36.0	34.3	37.4	35.5	37.1	35.2
M9	15:56:10	0.44	29.9	32.7	29.9	34.3	45.3	47.2	45.3
M10	16:02:40	1.25	40.2	61.0	55.9	65.2	63.8	71.3	61.4
M11	16:06:10	1.21	41.4	64.2	58.4	68.9	63.8	71.3	61.4
M12	16:13:10	1.55	39.5	63.6	59.0	67.5	65.7	73.2	63.5
				mean error:			0.73	3.59	
				R^2^:			0.74	0.76	
				mean error (ex. M4,M6,M9):			−0.79	2.37	
				R^2^ (ex. M4,M6,M9):			0.97	0.96	

**Table 6:** Parameters exported as colour maps.

Parameter	Unit	Description
**UTCI**	**°C**	**Universal Thermal Comfort Index** [29], perceived temperature/equivalent ambient temperature
**PMV**	**-**	**Predicted Mean Vote** according to Fanger [2] −3 = cold/0 = neutral/+3 = hot
**sMRT**	**°C**	**Sampled Mean Radiant Temperature** sampled with “human probe”, containing LW and SW components
**sMRT-LW**	**°C**	**Sampled Mean Radiant Temperature—LONGWAVE** sampled with “human probe”, considering only thermal radiation (LW)
**SW-diff**	**W/m^2^**	**Impact of shortwave diffuse component,** describing the impact on the sMRT considering all diffusely reflected or scattered solar components (sky, facades, grounds, vegetation)
**VF sky**	**%**	**Effective (physiologically weighted) sky view factor,** fraction of visible sky weighted by the angular sensitivity of the human model
**VF-veg**	**%**	**Effective (physiologically weighted) vegetation view factor,** fraction of visible vegetation weighted by the angular sensitivity of the human model
**VF-façades**	**%**	**Effective (physiologically weighted) façade view factor.** fraction of visible facades weighted by the angular sensitivity of the human model (facades are defined in a general way as surfaces with a slope > 30°)
**VF-ground**	**%**	**Effective (physiologically weighted) ground view factor,** fraction of visible ground weighted by the angular sensitivity of the human model (ground is defined as surfaces with a slope ≤ 30°)

## Appendix A

The following figures show the spatially resolved evaluation results listed in Table 6 in the form of colour maps.
